# Assessment of a Clothing Ensemble with an Active Heating Function Based on Thermal Manikin Tests

**DOI:** 10.3390/ma18235258

**Published:** 2025-11-21

**Authors:** Agnieszka Greszta, Magdalena Młynarczyk, Anna Dąbrowska, Sylwia Krzemińska, Monika Jangas, Łukasz Starzak, Paweł Marciniak, Bartosz Małachowski

**Affiliations:** 1 Department of Personal Protective Equipment, Central Institute for Labour Protection—National Research Institute, Wierzbowa 48, 90-133 Łódź, Poland; andab@ciop.lodz.pl (A.D.); sykrz@ciop.lodz.pl (S.K.); mojan@ciop.lodz.pl (M.J.); 2 Department of Ergonomics, Central Institute for Labour Protection—National Research Institute, Czerniakowska 16, 00-701 Warszawa, Poland; m.mlynarczyk@ciop.pl; 3 Department of Microelectronics and Computer Science, Lodz University of Technology, ul. Wólczańska 221, budynek B18, 93-005 Łódź, Poland; lukasz.starzak@p.lodz.pl (Ł.S.); pawel.marciniak@p.lodz.pl (P.M.); 4 Malachowski Alpine Workshop Bartosz Małachowski, Skoczowska 49, 43-426 Dębowiec, Poland; bartek@malachowski.pl

**Keywords:** active clothing, electrically heated garments, smart clothing, thermal comfort, thermal insulation

## Abstract

Heated clothing is an alternative to passive thermally insulating clothing used so far, made of thick, multi-layered fabric compounds. In this work, a personalized two-layer heated clothing ensemble for mountain rescuers was developed. It consisted of an electrically heated inner suit and an outer suit made of waterproof laminate. Total thermal insulation and local thermal insulations were determined using a thermal manikin. The heating system’s performance was assessed by comparing these results with those obtained with the heating turned off in the same ensemble, as well as with a down jacket added. It was confirmed that a thick thermally insulating layer (down jacket) can be eliminated through the application of electric heating. Heating improved the resultant effective thermal insulation of the clothing ensemble by 52% at a total power of 28.4 W. This exceeded the value obtained with the additional down jacket and no heating by 4%.

## 1. Introduction

### 1.1. Existing Electrically Heated Garment Solutions

Conducting long rescue missions in the mountains at low air temperatures, often during heavy rain and/or snowfall, or strong gusts of wind, requires wearing specialized protective clothing. Passive thermally insulating garments are unable to cope with the dynamically changing working conditions of mountain rescuers. In this situation, their thermal comfort can be improved with active clothing containing heating systems that even reduce the necessary thickness of passive insulating layers. Electronic control allows the thermal insulation of an active garment to be automatically regulated [1,2,3].

Electrically heated garments (EHGs, singular: EHG) usually consist of a textile clothing product with flexible heating parts in the form of pads, either permanently implemented in its structure or detachable (e.g., for washing)—normally including a sensor and/or a safety device, a control module, and an energy bank [4]. A flexible heating part combines a substrate material with an electrically conductive one which may be either metallic (e.g., metal fiber, metal-coated yarn, or metal nanowire) or non-metallic. The latter group includes carbon fibers, carbon nanomaterials (e.g., graphene or carbon nanotubes), and conductive polymers (e.g., polythiophene, polypyrrole, polyaniline, or polyethylenedioxythiophene) [4,5,6,7]. Flexible heating parts appear mainly in the form of heating foils or textiles [4].

There exist heating pads in the form of flexible foils where a conductive metal wire or carbon nanomaterial is immersed in, or mixed with, a polymer such as thermoplastic urethane, polyethylene terephthalate, polyimide, polydimethylsiloxane, or poly(methyl methacrylate). This structure ensures the stability of the conductive material and the safety of use. Other advantages include a very small thickness, short heat-up time, and uniform heat flux density distribution [4]. The main drawbacks are the lack of air and vapor/sweat permeability, and much lower flexibility in comparison with textile materials, all of which translate into a lower comfort of use.

Textile heating pads using metallic heating wires have been known for several decades. However, such wires are little resistant to bending, so they break easily, and they do not provide a uniform heat flux density distribution [8].

Some of the most popular heating materials presently used in EHGs are metal-based conductive yarns, whose mechanical properties are similar to those of textile yarns [7]. They are capable of quick generation of heat while being much less rigid than metal wires [9]. Conductive yarns made of stainless steel filaments [4], brass, silver-plated brass, copper, silver-plated copper, aluminum, or copper-clad aluminum are used [7,10]. It is also possible to combine metallic monofilaments with textile yarns such as cotton, polyester, polyamide, or aramid. This is usually achieved by embroidering or sewing [7,11], while alternative solutions include weaving and knitting [7,12]. A drawback of conductive yarns is that they easily produce short circuits, in particular, when the pad is folded. Protections are therefore often necessary, e.g., in the form of an insulating coating of the yarn or an additional insulating material [8,9].

Carbon fibers are another group of materials very often used in EHGs [4]. Their properties were summarized by Yang [13]. They show high emissivity (reaching 0.95) in far infrared and high heating power density even at low voltages (1.5 V to 12 V), which is favorable in terms of safety. Their ability to quickly heat up and cool down makes them suitable for precise and fast-acting temperature control.

In the case of linear heating elements, uneven heat flux density distribution is often a problem. A promising alternative is surface heating using materials obtained by coating [9,14]. A simple approach consists of coating a textile with a metallic nanomaterial, e.g., silver nanowires (AgNWs). It was shown in [14,15] that a cotton fabric coated with AgNWs heated up to 38 °C and 53 °C, respectively, when a voltage of 0.9 V and 1.2 V was applied, while the same fabric with a carbon nanotube (CNT) coating achieved a similar temperature only when the voltage was raised to 12 V [7]. Choi et al. [9] developed an innovative textile heating pad for clothing in which a fabric embroidered with electrodes was coated by screen printing with a composite paste containing multi-walled CNTs and a positive temperature coefficient carbon ink. A conductive yarn composed of silver and polyester threads was used for electrodes.

Heating part arrangement optimization aimed at the maximization of heating efficiency is an important and still topical research area [2,16,17,18]. Cho and Cho [16] fixed heating pads on the shoulders, abdomen, back, and waist. Based on skin temperature increase and subjective thermal sensations in a cold environment (−5 °C), they found that the location on the back was the most beneficial, followed by the waist, the abdomen, and shoulders. In the study conducted by Castellani et al. [17], at a low ambient temperature (−5 °C), heating the forearms had a beneficial effect on maintaining workers’ manual dexterity—as evidenced by improved fine motor skills—and grip strength. Kim et al. [18] compared three arrangements of heating elements: on the back, and on the back and forearms, as well as on the back, forearms, and the dorsal parts of hands. Their tests showed that simultaneously heating the back, forearms, and hands during a two-hour exposure to cold had a beneficial effect on body temperature and significantly improved hand dexterity and grip strength.

Kurczewska and Leśnikowski [19] developed a two-piece electrically heated clothing ensemble consisting of a jacket and trousers. The jacket was equipped with four heating pads, including two on the chest and two on the sleeves, while in the trousers, they were located at the height of the lower leg. The measurement and control system measured skin temperature, external temperature, and pulse to detect increased physical exertion. The system decided when to turn on or off the heating pads. Although the results of tests conducted in a cold store at −24 °C failed to demonstrate statistically significant differences in skin temperature between passive and active (heated) clothing, testers’ subjective perceptions indicated a significant improvement in their thermal comfort.

In 2008, Kolon Glotech (Seoul, Republic of Korea) launched a smart heated jacket where Heatex, the world’s first heating system based on a conductive polymer, was implemented in a thermal lining. That garment offered three heating levels (low or 40 °C, medium or 45 °C, and high or 50 °C). In addition, it had a built-in Global Positioning System (GPS) module for geolocation and a wind turbine generator, to be mounted on a sleeve, for supplying the GPS receiver and mobile devices [2].

Kayacan et al. [20] developed a heated vest with heating panels made of a knitted fabric containing stainless steel yarn. These were supplied with a battery pack using simple on–off control and were protected from short circuits with cotton cloth. They were 4 cm × 10 cm in size, two on the abdomen and two on the lower back. Only three-ply pads supplied with a Li-ion battery provided reasonably high heating levels for at least one hour.

Lee et al. [6] developed a smart heated jacket with heating pads made by embroidering, located on the back and in the hood. A textile switch on the arm and a mobile application were used as user interfaces for control, both providing the functions of switching the heating on and off, setting pad temperature, and checking the battery state. Additionally, the mobile application allowed individual heating zones to be selected.

Liu et al. [21] patented a heating system in clothing that consisted of a flexible heating fabric containing conductive yarn (metallic or metal-coated) and a temperature regulator. It was controlled by a smartphone and powered with batteries and photovoltaic cells.

The patent EP 3068189A1 [22] described a smart heated jacket where temperature and humidity were automatically regulated to values set by the user. One temperature sensor was in the armpit region, while another one, together with a humidity sensor, was inside the control unit.

Fan et al. [23] developed a heated jacket with an electronic temperature regulator controlled with a smartphone via a Bluetooth link. It was powered by a 5 V battery and had six heating pads located on the back, abdomen, and wrists, each one equipped with a thermistor for temperature sensing. Using a smartphone application, the user could set the temperature of the heating pads, with a maximum limit of 42 °C. If any irregularities were detected, heating was automatically stopped by a safety mechanism to avoid causing burns to the wearer.

Lee et al. [24] looked for the optimum skin temperature threshold for turning on and off heating pads by performing tests with human participants at 2 °C. On average, the testers turned on the heating when temperature dropped to 32.8 °C on the abdomen and 31.8 °C on the lower back, and they turned it off when temperature reached 48.8 °C and 40.1 °C, respectively. Moreover, the total heating time and the number of heating activations/deactivations were higher for women than for men, and women felt uncomfortable with the cold earlier than men.

### 1.2. Thermal Insulation of Clothing

Thermal performance of clothing is usually described by thermal insulation. This parameter is generally defined as the difference between the wearer’s skin surface temperature, T_s_, and the ambient atmosphere temperature, T_a_, divided by the resulting heat flux density (heat flow per unit area), h_c_, in the direction of the temperature gradient [25]:

(1) $$I=\frac{T_{s}-T_{a}}{h_{c}}$$

This quantity has the dimension of thermal resistance times area, corresponding to the SI unit of m^2^K/W, or K/(W/m^2^). The latter form shows that thermal insulation reflects the insulating properties of a clothing design independent of its surface area, thus enabling comparisons between garments of different sizes. Depending on elements included and test conditions, various values of thermal insulation may apply. They may be expressed in a dedicated clothing insulation unit, 1 clo = 0.155 m^2^K/W [26].

**Figure 1:**
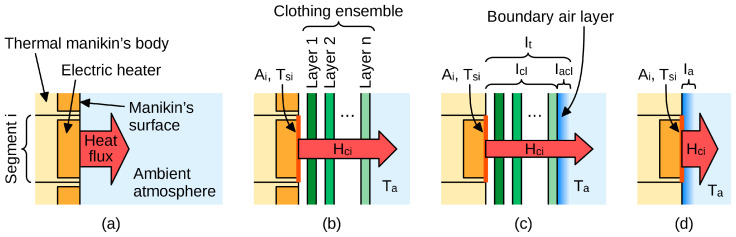
Heat flow in a test setup for thermal insulation employing a thermal manikin: (**a**) naked manikin section, idealized; (**b**) manikin wearing an n-layer clothing ensemble, idealized; (**c**) manikin wearing a clothing ensemble, with the boundary air layer shown; (**d**) naked manikin, with the boundary air layer shown.

To ensure standardization, repeatability, and reproducibility, thermal insulation of clothing is measured using a thermal manikin in a climatic chamber [25], which setup is schematically presented in Figure 1a. As heat flux density and surface temperature may differ from one body area to another, the manikin’s surface is divided into segments where these parameters can be considered invariable. Then, the total thermal insulation of a clothing ensemble is determined as follows [25] (see Figure 1b):

(2) $$I_{t}=\frac{\left(\left(\sum_{i}\frac{A_{i}}{A}T_{si}\right)-T_{a}\right)\times A}{\sum_{i}H_{ci}}$$

where A_i_ is the surface area of the i-th segment of the manikin, t_si_ is the temperature of this segment’s surface, A is the manikin’s total surface area, and H_ci_ is the heat loss flux (or heat flow rate) from the i-th segment.

Equation (2) results from the application of the so-called parallel calculation model. It is often preferred over the alternative serial model due to the lower accuracy of the latter when applied to a thermal manikin with homogeneous surface temperature [27]. Nevertheless, the serial model involves the evaluation of local thermal insulation [25] which can be used to describe the properties of a clothing ensemble in each segment separately:

(3) $$I_{ti}=\frac{\left(T_{si}-T_{a}\right)\times A_{i}}{H_{ci}}.$$

Total thermal insulation is influenced by the insulating effect of the boundary air layer adhering to the surface of the manikin or clothing (see Figure 1c,d). To assess the clothing ensemble alone, the thermal insulation of this air layer, I_acl_, must be determined and subtracted from I_t_. It is easy to measure the total thermal insulation of the boundary air layer adhering to a naked manikin, I_a_. This involves the same procedure as for I_t_, including the application of Equation (2), just with no clothing worn by the manikin, as illustrated in Figure 1d. The resulting parameter of the clothing ensemble is called effective thermal insulation and defined with the formula:

I_cle_ = I_t_ − I_a_. (4)

Some kinds of clothing must comply with specific standards, such as EN 342:2017 [28], that set requirements for resultant effective thermal insulation, I_cler_. The only difference between standard and resultant parameters is that the former are measured with the manikin stationary, while the latter, with the manikin moving its arms and legs. This applies to I_t_ and I_a_ alike, so

I_cler_ = I_tr_ − I_ar_ (5)

where I_tr_ is the resultant total thermal insulation of the clothing ensemble and I_ar_ is the resultant thermal insulation of the boundary air layer. Both these parameters are measured as presented in Figure 1c and Figure 1d, respectively, and evaluated using Equation (2), just with manikin’s limbs in motion.

Equation (4) assumes I_acl_ = I_a_. However, I_a_ corresponds to the total surface area of the manikin, A, while the outer surface of the clothing ensemble worn on the manikin, A_cl_, is larger, which makes the corresponding air layer insulation smaller. To account for this difference, clothing area factor is defined as

f_cl_ = A_cl_/A (6)

and used to calculate basic thermal insulation,

I_cl_ = I_t_ − I_a_/f_cl_. (7)

Wang and Lee [27] evaluated an electrically heated vest combined with a typical three-component clothing ensemble worn on a thermal manikin. The vest had a carbon polymer heating panel located on the back. At an ambient temperature of 0 °C, heating with a power of 5 W or 13 W increased the temperature of the vest itself, but it had an insignificant effect (of about 2%) on the total thermal insulation of the ensemble. Heating power had no apparent effect on the latter parameter evaluated using the parallel model. At −10 °C, vest temperature only increased after the power of 13 W was applied, and total thermal insulation of the ensemble insignificantly dropped.

Song et al. [29] compared the heating performance of an EHG with that of a chemically heated garment (CHG) using a thermal manikin at an ambient temperature of 2 °C. At an air velocity of 0.4 m/s, the tested EHG showed somewhat higher effective heating power and total thermal insulation: 0.365 m^2^K/W vs. 0.355 m^2^K/W for the CHG. Increasing the air velocity to 1 m/s considerably reduced thermal insulation, with no significant difference between the two garments.

Li et al. [26] compared the insulating properties of an EHG with and without an aerogel layer. The jackets evaluated had eight heating pads each, with a total power of 25.5 W, located on the upper arms, chest, abdomen, and back. Using a thermal manikin, thermal insulation of the clothing ensemble, effective heating power, and heating efficiency were measured for nine combinations of ambient temperatures (−5 °C, −10 °C, and −15 °C) and air velocities (0 m/s, 0.5 m/s, and 1 m/s). Heating increased the thermal insulation from 0.304 m^2^K/W (for the same ensemble with the heating off) to 0.326 m^2^K/W for the garment without aerogel and from 0.330 m^2^K/W to 0.381 m^2^K/W for the one with aerogel. Variations in ambient temperature had negligible effect on these values. On the other hand, increasing air velocity from 0 m/s to 1 m/s resulted in a decrease in thermal insulation of between 0.09 m^2^K/W and 0.14 m^2^K/W. Both lowering ambient temperature and increasing air velocity decreased effective heating power and heating efficiency of the ensemble.

Park et al. [30] investigated how placing heating pads in different clothing layers affects thermal insulation. Pads mounted on a T-shirt provided an effective thermal insulation of 0.331 m^2^K/W (at an ambient temperature of −5 °C), while it dropped to 0.315 m^2^K/W after they were moved to the fourth layer (a down jacket). With the heating pads in the underwear, the effective thermal insulation of the clothing ensemble was 5% to 7% higher than without any heating.

### 1.3. Motivation and Scope of This Work

Heated clothing solutions present on the market are insufficient to meet the requirements of the demanding working conditions of mountain rescuers. In particular, they offer too-low heating power and too-low heating efficiency—one of the causes being too-small heating pad areas—or do not adjust properly to wearers’ body shapes. Dedicated EHGs could also improve thermal comfort of other professionals working outdoors. However, research papers published so far have only studied general-purpose clothing intended for personal use, while professional garments must meet the requirements of relevant standards such as [28]. Finally, most studies have considered heating only globally, with the same power applied in all pads, while heating needs are different across body regions.

For the above reasons, we developed a personalized, complete heated clothing ensemble for mountain rescuers, including a heated suit and an outer suit made of waterproof laminate, as well as an optional down jacket for extreme conditions. The heated suit enabled heating power in four body zones to be set independently. The structure of the prototype ensemble will be described in detail in Section 2.1.

The aim of this study was to assess the heating system in this ensemble by performing tests on a thermal manikin in a climatic chamber according to the procedure specified in Section 2.2, with results shown in Section 3. The parameters measured to evaluate heating performance included total thermal insulation, I_t_, as the basic parameter for comparisons; resultant effective thermal insulation, I_cler_, for compliance assessment; and basic thermal insulation, I_cl_, for comparison with other works. They will be discussed in Section 4.1.

Until now, values measured locally in each manikin section, such as local heat flux, H_ci_, have been used by authors only to evaluate thermal insulation of entire clothing ensembles according to Equation (2). While this provides a single value convenient for comparisons of accomplished solutions, it gives no insight into the need for or performance of individual heating pads. For this reason, we additionally determined local thermal insulation in each manikin segment. These results will also be presented in Section 3, and their relevance and utility will be demonstrated in Section 4.2.

Final conclusions will be formulated, and future research plans will be presented in Section 5.

## 2. Materials and Methods

### 2.1. Tested Object

The object of the tests was a personalized active protective clothing ensemble with a heating function, developed for mountain rescuers. It consisted of the following garments:

- an electrically heated suit (inner layer),
- a down jacket (middle layer, optional),
- an outer suit made of waterproof laminate (outer layer).

These can be arbitrarily combined depending on weather conditions and individual user preferences. Table 1 provides key information on the materials used in each garment, including surface mass, thermal resistance, and water vapor resistance measured in this work.

**Figure 2:**
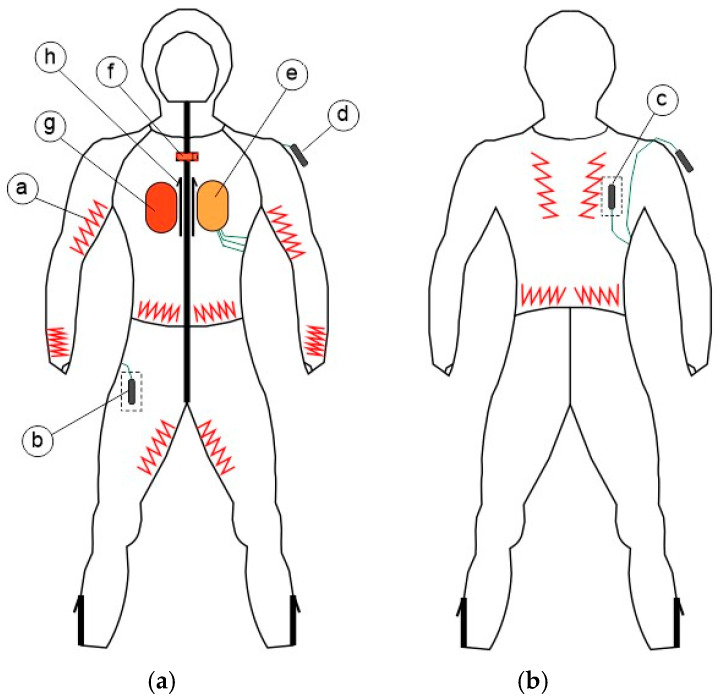
Heated suit diagram: (**a**) front view; (**b**) back view (a: heating pad, b, c: undergarment microclimate temperature sensors, d: ambient temperature sensor, e: controller, f: metal snap, g: battery pack, h: zippers).

As illustrated in Figure 2, the heated suit was equipped with

- twelve flexible heating parts (pads) grouped into four independently controlled heating zones: arms, legs, abdomen, and back, with a total surface area of 840 cm^2^ (a),
- two undergarment microclimate temperature sensors (b, c) and one ambient temperature sensor (d),
- a controller with buttons, onboard accelerometer, gyroscope, and pressure sensor, as well as an attached undergarment temperature and humidity sensor (e),
- a metal snap (f) and
- a battery pack (g).

Sensors have been included in this list for completeness, though they were not used during the tests presented in this paper. In addition, a mobile application for smartphones and smartwatches that executed high-level control functions and provided a user interface was developed.

The heated suit was made of elastic polyester knitted fabric with a microfleece inner layer to ensure a tight fit to the user’s body. Heating pads were implemented on its inside to reduce the number of insulative layers between the user’s skin and pads. The exact locations of the latter were chosen with consultation with mountain rescuers and aligned with arterial pathways to increase the rate of heat transfer within the body and, consequently, reduce the number of pads needed.

**Figure 3:**
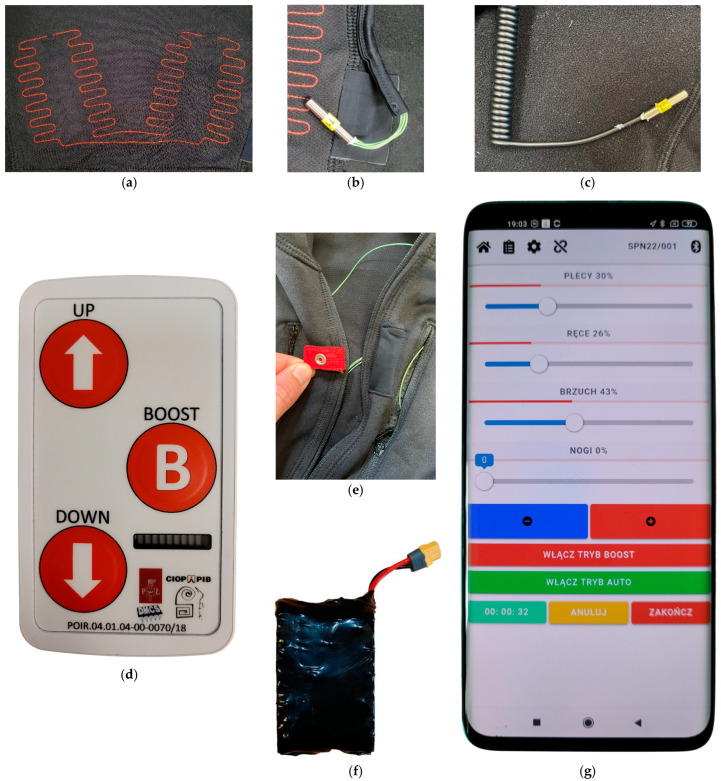
Heating system elements: (**a**) heating pad (back view), (**b**) undergarment temperature sensor, (**c**) ambient temperature sensor, (**d**) controller, (**e**) metal snap, (**f**) battery pack, (**g**) mobile application for smartphones (home screen).

The heating pads (Figure 3a) were produced using a computerized numerical control lockstitch sewing machine RPAS-LM(HM)-R-1-900×600-A-IS2-VR2-LH50,RH360 (Tianjin Richpeace AI, Tianjin, China). The conductive thread was sewn onto the substrate fabric according to a programmed pattern. To connect these heating elements to the controller, thin, flexible copper wires with an electrical resistance of 0.18 Ω/m were used. The heating thread was joined with these wires using copper sleeves. To ensure protection against short circuits, the heating pads were glued to the suit material with a thermoplastic polyurethane adhesive strip.

Inside the suit, the undergarment temperature sensors (Figure 3b) were fixed on the right thigh and the right scapula (Figure 2, items b and c). The ambient temperature sensor (Figure 3c) was output through an opening in the stand-up collar, to be attached to the outermost clothing layer (Figure 2, item d). At the front, a metal snap (Figure 3e) was added to the collar (Figure 2, item f). It was connected with the heating system in a way that requires the former to be fastened to enable the activation of the latter. This prevents accidental activations after the user takes off the suit, which in extreme cases, could lead to the suit catching fire, e.g., due to two heating pads coming into contact.

The controller (Figure 3d) was an embedded electronic system whose structure and operation were described in [31]; it combined control, monitoring, and power supply functions. It supplied electric power to the heating pads combined into six pairs that were subsequently grouped into the four heating zones. The maximum total power was approximately 51 W and the controller’s mass was ca. 140 g. The custom-made lithium-ion battery pack (Figure 3f) had an estimated end-of-life capacity of ca. 51 Wh and a mass of ca. 250 g.

It was possible to increase or decrease heating power globally using buttons on the controller or individually for each zone using the mobile application (Figure 3g). The values introduced in the latter were transmitted to the controller via a Bluetooth connection.

### 2.2. Testing Methodology

**Figure 4:**
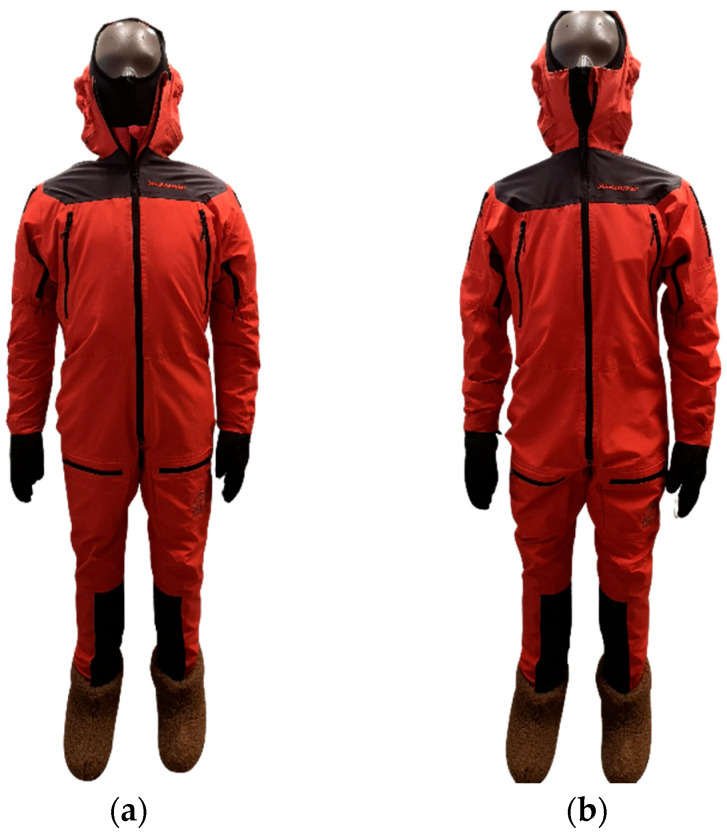
The thermal manikin used, dressed for the tests: (**a**) clothing ensemble with the down jacket (the DJ configuration); (**b**) clothing ensemble without the down jacket (the REF, HL and HH configurations).

To assess the performance of the heating system, thermal insulation tests were conducted for four different clothing ensemble configurations specified in Table 2. The heating powers applied have been listed in Table 3. The ensemble was tested in combination with gloves, warm socks, and shoes, as shown in Figure 4.

As required by the EN 342:2017 standard [28], before the tests, the garments had been subjected to 20 cleaning cycles according to EN ISO 6330:2021 [32] in the conditions specified in Table 4. Biały Jeleń detergent (POLLENA Kosmetyki i Mydła Naturalne, Ostrzeszów, Poland) was used to wash the heated suit and the down jacket, and the reference detergent 3 (ECE 98) (James H. Heal & Co. Ltd., Halifax, UK) was used for the outer suit. Procedure A of drying consisted of hanging each hydro-extracted garment unfolded, suspended from a line in the direction of use, in still air, under ambient conditions. Procedure F involved placing the garment in a tumble dryer (Type A1, vented) together with an appropriate ballast (Type III, 100% polyester) and tumble-drying it at the specified temperature according to Clause 10.4.3 of [32].

**Figure 5:**
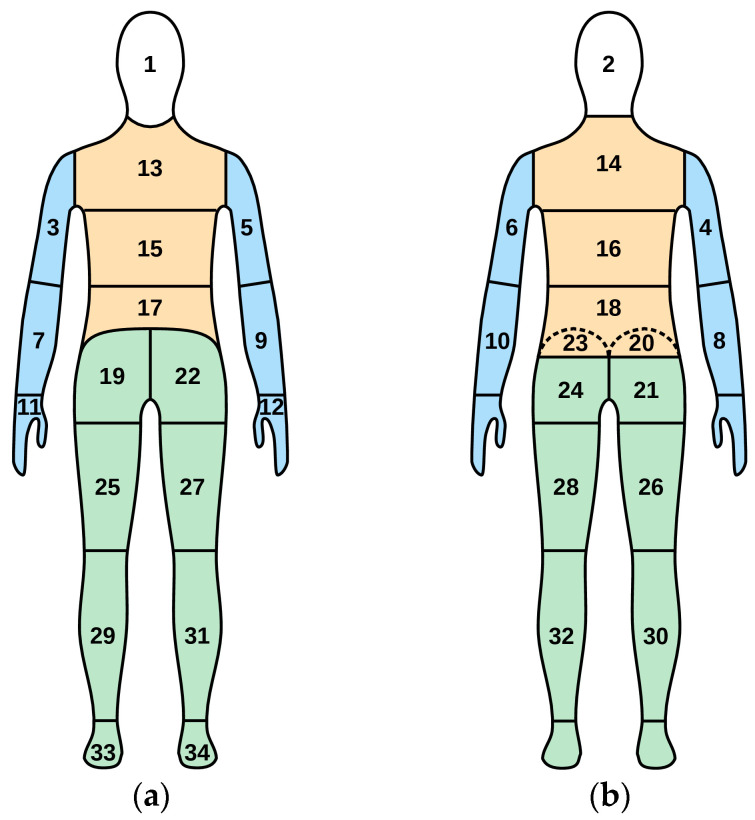
The thermal manikin used with individual segments and regions marked (blue: upper limbs region; orange: torso region; green: lower limbs region; see Table 5 for explanations of numerical symbols): (**a**) front view; (**b**) back view.

The tests were performed in accordance with the EN 342:2017 [28] and EN ISO 15831:2004 [25] standards in a Weiss WK23 climatic chamber (Weiss Technik, Reiskirchen, Germany). Climatic conditions inside, including ambient temperature, T_a_, were monitored with two microclimate meters: INNOVA 1221 (LumaSense Technologies, Ballerup, Denmark) and Delta OHM HD32.1 (Delta OHM, Caselle di Selvazzano, Italy). The manikin used was Thermetrics Thermal Manikin—Newton (Measurement Technology Northwest, Seattle, WA, USA), consisting of 34 segments (see Figure 5 and Table 5). Segments 20 and 23 were hidden as the manikin was standing, so the corresponding data were not included within the results presented in Section 3. The constant surface temperature operating mode was selected and the temperature was set to 34 °C as prescribed in [25,33]. The heat flux from each segment, H_ci_, was automatically adjusted by the manikin’s control system as required to keep this segment’s surface temperature, T_si_, at the set value.

**Figure 6:**
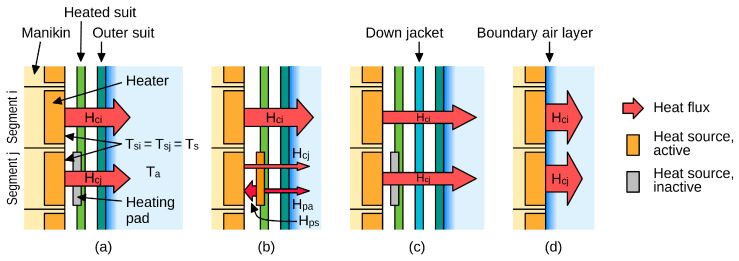
Simplified test setup arrangements for measuring total thermal insulation, I_t_: (**a**) reference configuration (REF: no jacket, heating off); (**b**) heated clothing with no jacket (HL, HH); (**c**) unheated clothing with the down jacket (DJ); (**d**) naked manikin (measurement of the total thermal insulation of the boundary air layer, I_a_).

The different thermal manikin and garment arrangements applied throughout tests have been presented in Figure 6. In the reference case (the REF configuration, Figure 6a), heating pads supply no heat. Hence, the only heat fluxes are those produced by the heaters inside the manikin to maintain the surface temperature of T_s_ under the current ambient temperature, T_a_. Heat flux densities from segments covered by heating pads (such as H_cj_ in Figure 6a) are smaller than those from the remaining ones (such as H_ci_) due to the additional insulation introduced by the pads. From the individual heat fluxes, H_ci_, measured by the manikin, the total thermal insulation of the clothing ensemble, I_t_, is calculated using Equation (2) and local thermal insulation in each segment, I_ti_, is calculated from Equation (3).

When the garment heating is on (the HL and HH configurations, Figure 6b), each heating pad produces a heat flux towards the manikin’s surface, H_ps_. The latter decreases the heat flux H_cj_ that the relevant segment’s heater must supply to keep its surface temperature, T_sj_, at the set level, T_s_. This increases local thermal insulation in accordance with Equation (3) and the resulting total insulation according to Equation (2). Figure 6b also shows that a portion of the heat produced by the pad is transferred to the ambient air, giving rise to the heat flux H_pa_. The latter increases the electric power consumption by the system.

With the down jacket added and the garment heating off (the DJ configuration, Figure 6c), the situation is like that of Figure 6a except that all heat fluxes are smaller. Finally, Figure 6d shows the manikin naked, as configured for the measurement of the total thermal insulation of the boundary air layer, I_a_, which is still calculated from Equation (2).

To assess the compliance of the clothing ensemble with the thermal insulation requirement of [28], the resultant effective thermal insulation of the former, I_cler_, must be calculated from Equation (5). This requires the resultant insulations I_tr_ and I_ar_ to be determined. According to [25], they should be measured with the manikin moving its limbs at the rate of 45 double steps per minute cross walking, corresponding to the walking speed, w, of 0.95 m/s. However, such tests would require the tested clothing ensemble to be damaged by cutting holes for the manikin’s hooks. To avoid this, the parameters concerned were calculated by applying a correction formula to total thermal insulations, I_t_ and I_a_, as provided for in the 2004 edition of EN 342 [34]:

(8) $$I_{tr}=I_{t}\times \left(0.54\times e^{\left(-0.15v_{a}-0.22w\right)}+0.5\right)$$

where v_a_ is the air velocity in m/s and w is the walking speed in m/s. For the boundary air layer, I_tr_ and I_t_ were replaced with I_a_ and I_ar_, respectively.

In line with [25], air velocity in the climatic chamber, v_a_, was set to 0.4 m/s, while air temperature, T_a_, was adjusted so that heat flux from each segment of the manikin was no less than 20 W/m^2^; this condition was met at −7 °C. The heating function of the clothing ensemble was initially inactive. Once a steady state was reached in these conditions, the mobile application was started. In the case of the HL and HH configurations, heating powers were then set according to Table 3, and a new steady state was attained.

For the HH configuration, the individual zone heating powers shown in Table 3 were selected as the highest that still resulted in positive heat flux from each manikin segment. This was because the manikin used did not support negative heat flux, i.e., its surface being effectively heated from the outside, which can happen at high heating power. When the heating power of each pad was set to its maximum, the heat fluxes from the manikin segments directly affected by heating pads (i.e., those on the back, arms, and abdomen) were all zero, making measurements impossible. This was due to the heaters inside these segments having automatically switched off as the surface temperature there exceeded the set value of 34 °C.

As soon as heat fluxes and temperatures ultimately stabilized in all segments, measurements were taken. In addition to total thermal insulation of the clothing ensemble, I_t_, calculated from Equation (2), local thermal insulation in each manikin segment, I_ti_, was evaluated from Equation (3). Based on the former, as well as on results obtained with the manikin naked (I_a_), basic thermal insulation, I_cl_, was determined using Equations (7) and (8), and resultant effective thermal insulation, I_cler_, was found from Equation (5).

## 3. Results

### 3.1. Thermal Insulation

The thermal insulation values obtained for the four tested clothing configurations have been presented in Table 6. The reference (REF) configuration (no down jacket, heating off) has been the basis for calculating the relative results. Total, basic and resultant effective thermal insulation of the high heating power (HH) configuration (no down jacket, maximum heating powers allowed by the manikin) were even slightly higher than those of the down jacket configuration with the heating off (DJ). In both these cases, the resultant effective thermal insulation was about 50% higher than the reference one.

In [28], resultant effective thermal insulation is one of the principal parameters for performance assessment of protective clothing against cold intended for ambient temperature of −5 °C or less. The universal lower limit of 0.265 m^2^K/W was only exceeded by the DJ and HH (28.4 W total heating power) configurations. The heating power of 10.3 W applied in the HL configuration was insufficient to meet the requirement of [28], despite increasing thermal insulation by 15% compared to the reference case.

### 3.2. Local Thermal Insulation

**Figure 7:**
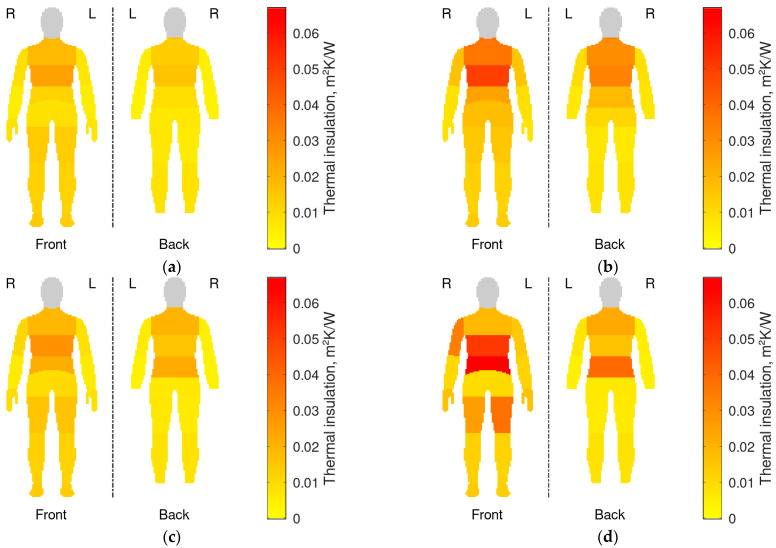
Local thermal insulation of clothing parts corresponding to manikin segments, for different clothing configurations (L: left, R: right; gray: segments not analyzed in this work): (**a**) REF; (**b**) DJ; (**c**) HL; (**d**) HH.

**Figure 8:**
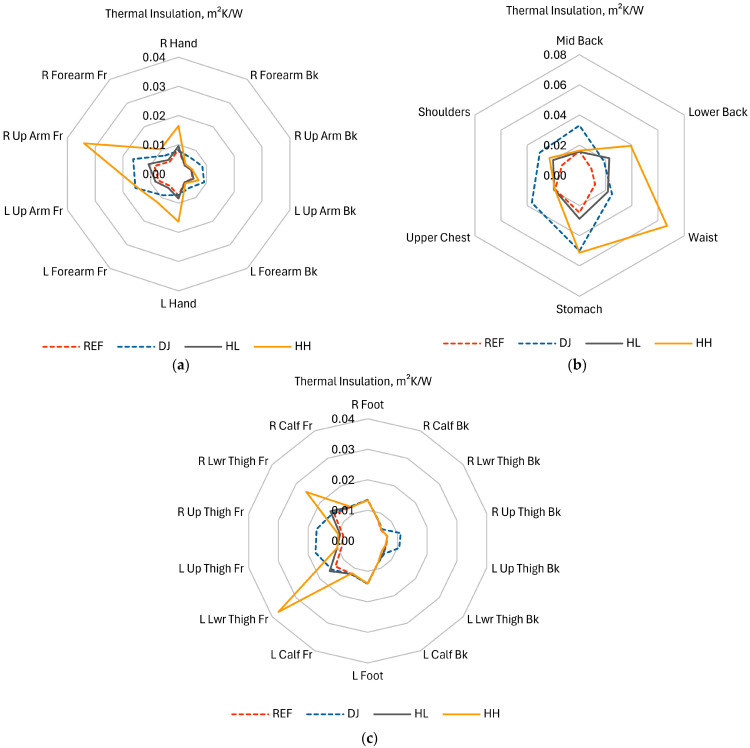
Local thermal insulation, for different clothing configurations, of clothing parts corresponding to manikin segments located on (**a**) upper limbs; (**b**) the torso; (**c**) lower limbs.

**Figure 9:**
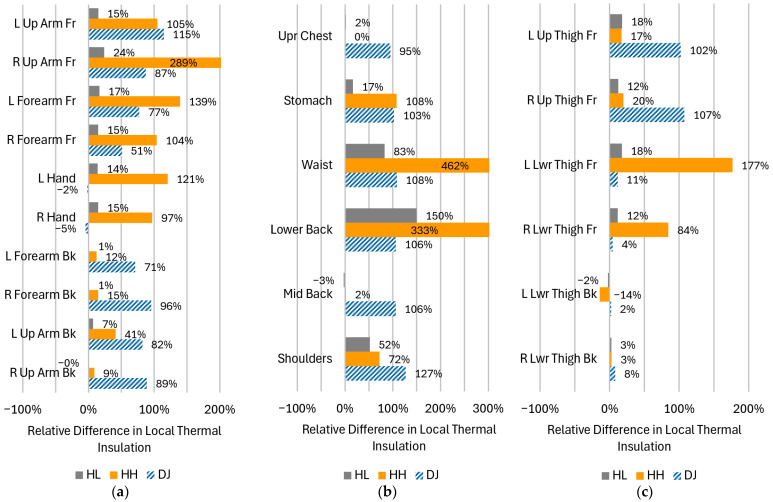
Relative difference in local thermal insulation between different clothing configurations and the reference configuration (REF), of clothing parts corresponding to manikin segments located on (**a**) upper limbs; (**b**) the torso; (**c**) lower limbs.

Local thermal insulations, I_ti_, of individual clothing parts covering the corresponding manikin segments are presented in Figure 7 for the four configurations tested. To ease quantitative comparisons, the same data have also been plotted using radar graphs in Figure 8. The corresponding relative differences with respect to the reference case (the REF configuration: no down jacket, heating off) have been plotted in Figure 9.

Based on these results, the following are generally true.

- The effect of heating (HL and HH) was greatest in segments where heating pads were located (compare Figure 7c,d against Figure 2 and Figure 7a). This is particularly apparent on the waist (Figure 8b), as well as on the front of the right arm (Figure 8a, R Up Arm Fr) and lower thighs (Figure 8c, R/L Lwr Thigh Fr).
- As a rule, the impact of heating was more prominent on the front than on the back. This is directly represented in Figure 7c,d and Figure 9, as well as reflected in the offset of centers of mass of shapes in Figure 8. The upper back (Shoulders) was the only exception.
- The effect of the down jacket (DJ) was more uniform than that of heating (HL and HH) not only in terms of different segments but also front vs. back side (as seen in Figure 7b in comparison with Figure 7d as well as in Figure 9). Still, Figure 7a,b and Figure 8 reveal some bias towards the front of the body in terms of absolute values for both configurations with the heating off (DJ and REF).
- When operated at the higher power (HH), the heating system tended to outperform the down jacket (DJ) on the front, while the opposite was true on the back.
- Low heating power (HL) had a small (still observable) impact even at the front, with increase in local thermal insulation ranging from 10% to 20% in most segments.

When body regions are considered separately, additional detailed observations can be made, some of which are against the general trends described above.

1. Upper limbs
  - With the heating on, whatever the power, the highest relative increase in local thermal insulation was recorded in the right arm front segment (R Up Arm Fr): by 289% with the HH configuration and 24% with the HL one. In both cases, the heating effect was much smaller in the corresponding segment of the left limb (L Up Arm Fr), which suggests that this was due to a worse adhesion of the left pad to the manikin.
  - The effect of the heating system was stronger on the front, where local thermal insulation increased by more or nearly 100% in all segments at high power (HH). This was more than with the down jacket (DJ, 77% to 115%), except on the problematic left upper arm. On the other hand, the much weaker effect of the down jacket on the right forearm (R Forearm Fr, 51%) can be due to how the jacket was arranged on the manikin.
  - On the back, the impact of the heating system was close to none at low power (HL) and still low at high power (HH, 9% to 41%). Conversely, the effect of the down jacket (DJ) was similarly strong on the back as on the front (between 71% and 96%). On the other hand, the down jacket had no impact on hands, where the application of heating increased the local thermal insulation like in the front segments of the arms (97% and 121%).
2. The torso
  - It is only in this region that the effect of the heating system was strong even at low power (HL). It reached 83% on the waist and 150% on the lower back, the latter result exceeding that of the down jacket (DJ, 108%). It was also in these segments that the strongest effect over the entire body was observed at high power (HH): an increase in thermal insulation of 462% (up to 0.067 m^2^K/W) in the waist area and of 333% (up to 0.039 m^2^K/W) in the lower back one. This was where heating pads were located, but a substantial increase (108%) occurred also on the abdomen (Stomach).
  - When operated at high power (HH), the heating system outperformed the down jacket (DJ) in the lower part of the torso, was on par with it on the abdomen and only worse on the shoulders. On the other hand, the heating had no effect on the mid back and upper chest; in these segments, the uniformity of the jacket’s effect (improvements in local thermal insulation between 95% and 127% over the torso) proved crucial.
3. Lower limbs
  - The positive impact of the heating system was only noted on the front, consistently with the positioning of the leg pads (compare Figure 7d with Figure 2). This impact was only significant on lower thighs and at the higher power (L/R Lwr Thigh Fr; HH), with an increase in local thermal insulation of 177% (left) and 84% (right). In these segments, the down jacket (DJ) performed worse (11% and 4% increase) than low-power heating (HL; 18% and 12%, respectively); this was because the jacket did not extend to these areas.
  - Conversely, the jacket improved local thermal insulation of the upper thighs (L/R Up Thigh Fr) which it covered, like elsewhere (by 102% and 107%). The heating system performed poorly in that area (17% and 20% improvement at high power), because pads were located lower. Still, heat transfer from neighboring pads is seen. It must be noted that the leg pads operated at full power in the HH configuration (Table 3).
  - None of the tested clothing configurations were able to increase local thermal insulation on the back of lower thighs where pads were missing and the jacket did not reach.

## 4. Discussion

### 4.1. Overall Thermal Insulation

The use of the heating function in the inner suit improved protection against cold. This is indicated by the much higher total and resultant effective thermal insulation (by 38% and 52%, respectively) in comparison to those of the same ensemble with the heating off (see Section 3.1 and Table 6). However, heating pads had to be supplied with sufficient electric power to achieve thermal insulation on par with that provided by the down jacket as an extra passive layer. The values applied in this work (4.8 W on the back, 12.9 W on the arms, 2.6 W on the abdomen and 8.6 W on the legs) proved sufficient in this respect and even permitted the heating system to outperform the jacket (by 3% and 4% in terms of total and resultant effective insulation, respectively).

The clothing ensemble developed in this work has been compared to similar prototypes in Table 7. Its total thermal insulation at the heating power of 28.4 W (0.379 m^2^W/K) was similar to that obtained by Song et al. [29] at 26.5 W (0.365 m^2^W/K). Although these results were obtained at different ambient temperatures, this was earlier found to have no significant effect [26]. However, the beneficial effect of heating was remarkably stronger in the clothing ensemble developed in this work, as it increased total thermal insulation by 38% (with respect to the same ensemble unheated), compared to 11% obtained in [29] with a power only 4% smaller. On the other hand, the same relative increase of 11% was achieved in this work using a heating power 2.6 times lower (10.3 W).

Wang and Lee [27] studied an ensemble composed as in [29], though with the heated vest used as a middle layer instead of an outer one. Its total thermal insulation of 0.26 m^2^K/W was much lower than in both [29] and our work. This can be traced to just a single heating part having been applied, resulting in a small total heated area.

The results published by Li et al. [26] are ambiguous as to what parameter they represent. Although Figures 6 and 8 there show exactly the same values at zero air velocity, the former is labeled and referred to as “I_cl_” (thus basic insulation), while the latter, as “I_t_” (suggesting total insulation). Both quantities have therefore been listed for our ensemble in Table 7. Whatever the interpretation, the effect of heating was stronger in this work than in [26], even at a power 2.4 times lower (11% or 15% increase at 10.3 W vs. 7% at 25.2 W); the corresponding differences at a similar power (28.4 W) were obviously larger (38% or 50%). Relative increase was greater for basic insulation, because—according to Equation (7) and in contrast with total insulation—the former does not include the insulation of the boundary air layer, which is unaffected by heating.

The results in Table 7 suggest that total heated area is not of primary importance for thermal insulation. The difference in the latter parameter between [26,29] is not large assuming [26] gives basic insulation. In our work, a similar basic insulation of 0.254 m^2^K/W corresponded to a total insulation of 0.305 m^2^K/W. If the latter value was supposed for [26], total insulation there would be only 16% lower than in [29], while total pad area was 37% smaller and air velocity was 25% higher (which should decrease thermal insulation even more, according to [26]). The hypothesis of a weak influence of total heated area is further supported by the results obtained for our ensemble where the total pad area was 2.6 and 1.7 times smaller than in [29] and [26], respectively, still thermal insulation was higher and the effect of heating was stronger.

The higher efficiency of our solution must therefore be due to a combination of the following other structural differences that can be identified in Table 7.

1. The number of heating pads was the highest in our EHG. Extra pads were located in body areas unheated in [26,29]: lower arms, upper legs (instead of knees as in [29]), and upper arms (pads missing there in [29]).
2. Heating pads were integrated within the innermost clothing layer as compared to the outer one in [26,29], or the middle one in [27]. The relevance of this factor was already noted in [30].
3. Sewn-on steel thread was used as the heating element instead of carbon wire [29], graphene sheets [26] or carbon polymer fabric [27]. It is remarkable that all these less efficient solutions use carbon-based materials. It can also be noted that the best of them, i.e., the one described in [29], has the heating element in the form of a wire applied on a polyester substrate. As these characteristics are shared with our EHG, it is probable that they also played a role. This may also be the cause of thermal insulation being greater in [29] than in [26] despite the pads being closer to the body in the latter solution.

### 4.2. Local Thermal Insulaton

Local thermal insulation has not been studied in the literature so far. Although it is less convenient than single synthetic indicators such as total thermal insulation, it provides valuable information that can be crucial for the optimization of a clothing ensemble or its adaptation to specific purposes and use conditions. These may include resizing (in terms of both surface area and power) and rearranging heating pads to reduce the cost and improve ergonomic properties while not compromising the wearer’s thermal comfort.

The greatest increase in local thermal insulation was recorded in these manikin segments where heating pads were located (Section 3.2 and Figure 7c,d, in comparison with Figure 2 and Figure 7a), which could be reasonably expected. The heating system had the greatest impact on the waist and lower back segments (Figure 9), which coincided with the locations of the largest heating pads while their supply power was relatively low (Table 3). This suggests that individual pad area is an important factor. The increase in thermal insulation was also substantial (though lower) in the abdomen (Stomach) and shoulders segments. Generally, the highest local thermal insulation was measured (even with an unheated clothing ensemble; Figure 7 and Figure 8) and the lowest heating power was applied (Table 3) on the torso. All these facts combined indicate that heating needs are the lowest there.

On limbs, local thermal insulation (Figure 7 and Figure 8) as well as its relative increase (Figure 9) were higher on the front, which was due to the placement of the relevant heating pads. This is confirmed by the results for the torso, where thermal insulation increased greatly in two segments on the back: Lower Back and Shoulders, where pads were located, while it did not change in the Upper Chest one, where they were missing. The results for the torso were consistent with those presented in [16] in that the pads on the lower back and waist were the most efficient ones, though in reverse order.

A significant increase in local thermal insulation was also noted on the abdomen (Stomach). It can be hypothesized that this was an effect of heat transfer from the pads on the waist as well as extra heat generation and separation from the environment by the controller and battery pack. Undoubtedly, heat transfer from adjacent heated segments extended to the front of the upper thighs (L/R Up Thigh Fr), the back of upper limbs (L/R Forearm/Arm Bk), and hands. In regard to hands, this was consistent with the findings published in [18]. The effects of heat diffusion were limited to 20% of added thermal insulation as compared to 72% at minimum—and usually more than 100%—in segments heated directly. Hands were a notable exception, with relative increase of 121% and 97%.

Differences between some corresponding front right and left segments can be noticed in Figure 7d and Figure 8a,c, e.g., on arms (R vs. L Up Arm Fr) and lower thighs (R vs. L Lwr Thigh Fr). In the former case, insulation change (Figure 9a) was unusually high on the front right (289%) and back left (41%), while being low on the back right (9%) and front left (105%). Based on observations, it can be hypothesized that this resulted from the sleeves having been twisted when dressing up the manikin. This may have displaced the heating pad on the right arm to the front, and the one on the left arm, to the back. Moreover, the greater increase in thermal insulation on the front right might be due to heat dissipation in the nearby battery (see Figure 2). In the case of thighs, it can be speculated that the difference observed was caused by uneven tightness-of-fit of the garment to the manikin, which gave rise to free spaces filled with air. Repeated tests with improved control of clothing fit would be required to confirm the statistical significance of these asymmetries.

The importance of local insulation as a parameter can be demonstrated when the results for the heated ensemble (HH) are compared with those for the unheated one with the down jacket (DJ). Although their total, basic and effective thermal insulations were similar (Table 6), local thermal insulations (Figure 7 and Figure 8) indicate that the down jacket provided much more uniform protection against cold in the areas it covered. This was owed to the down insulation being present all over the jacket, in contrast to heating pads in the suit. On the other hand, the suit outperformed the jacket in all but one section where pads were located, as well as on hands.

Despite the uneven heat distribution in the heated suit, the number and area of heating pads must be limited by ergonomic considerations. Their introduction to a garment inevitably reduces its flexibility and elasticity even if modern materials and technologies are employed. Pad arrangement in the developed heated suit was based on consultations with prospective end users, and it improved thermal insulation in areas not affected by the alternative down jacket (hands and lower thighs).

## 5. Conclusions

A two-layer clothing ensemble for protection against cold intended for mountain rescuers was developed, composed of an inner heated suit and an outer waterproof suit. An optional down jacket can be used as a middle layer in extremely unfavorable weather conditions such as low temperature, strong wind, or high humidity.

The tests carried out using a thermal manikin in a climatic chamber provided data on local heat fluxes from the manikin’s segments. On this basis, local, total, basic, and resultant effective thermal insulation were determined according to the EN ISO 15831:2004 [25] and EN 342:2004 [34] standards. When operated at a total power of 28.4 W, the heating system increased the resultant effective thermal insulation of the developed clothing ensemble from 0.183 m^2^K/W to 0.279 m^2^K/W, or by 52%. This was even slightly better than achieved after the extra down jacket was added as a third layer while the heating function was deactivated (0.267 m^2^K/W).

Both the heated two-layer ensemble and the unheated three-layer one met the thermal insulation requirement of EN 342:2021 (0.265 m^2^K/W) [28]. This demonstrates that a thick insulating layer (a down jacket in this case) can be eliminated by adding a heating function to a much thinner inner suit, without compromising the insulating properties of the clothing ensemble, while allowing greater freedom of movement. Supplying heating pads with sufficient electric power proved vital for this achievement, as with a total power of 10.3 W, the performance of the same ensemble was just 20% better (0.211 m^2^K/W) than with the heating off and the relevant requirement of EN 342 was not met in this case.

In terms of total thermal insulation, the developed clothing ensemble performed similar to, or better than, those presented by other authors. At a total heating power of 10.3 W, that parameter was 13% higher than measured in [27] at 13 W. At 28.4 W, 0.379 m^2^K/W was achieved with our ensemble, which is consistent with 0.365 m^2^K/W shown in [27] for 26.5 W (both values being 4% lower than in this work). However, the effect of the heated layer alone was stronger in our clothing, as it produced a relative increase in total thermal insulation identical to the one obtained in [29] (11%) when powered with just 10.3 W, or 2.6 times less than applied in that study.

The results of the analysis of structural differences between various solutions suggest that total area of heating pads is less important than their number, distribution over the entire body, and integration into a clothing layer as close to the body as possible. The heating element itself seems to affect the resulting thermal insulation even more than pad placement. A systematic parametric study using multiple prototypes would be required to corroborate these speculations. Among the analyzed solutions, conductive threads or wires applied on a substrate outperformed conductive sheets or fabrics, and steel performed better than carbon-based materials, which suggests that traditional heating technologies remain more advantageous than novel ones.

It should be noted that there was an unused reserve of heating power in the presented clothing ensemble, as all heating pads, except for those located on thighs, operated at just 25% to 75% of their full power. This allows the heating system to be optimized in the future, e.g., by decreasing the power of selected pads, shifting them to other areas, or reducing the size and weight of the battery pack.

While not analyzed by other authors, local thermal insulation in particular segments are vital in the optimization process of a heated suit as it provides a better understanding of individual heating pad contributions to total thermal insulation. For example, these data revealed that the heat generated by the forearm pads effectively propagated to the hands. This confirmed the findings of [17] and is beneficial in view of the intended application of the developed clothing ensemble, where hand dexterity and grip strength are of prime importance. Heat propagation from the front to the back of upper limbs as well as from the lower to the upper parts of thighs was also observed, but not so prominent. Moreover, the abdomen was well-protected from cold even though no heating pads were present there. Conversely, the heating had no effect on lower and back parts of legs. Inertial heat transfer after the heating function is turned off requires further investigation.

The strongest impact of the heating system was obviously observed in these manikin segments where pads were located. Positive correlation between individual pad area and local thermal insulation was noticed. Local thermal insulation values also suggest that the controller and the battery pack may have influenced heat exchange in the system. The use of a thermal imaging camera is planned throughout future tests to confirm or rule out this supposition.

The down jacket provided more uniform protection against cold than the heated suit. However, it should be noted that—in contrast to the latter—the jacket had no effect on those body regions that it did not reach. Moreover, it seems possible to extend the beneficial effect of the heated garment to the bottom of legs, probably even including feet, as suggested by the results obtained for hands.

It must be emphasized that while being a useful tool in assuring repeatable test conditions and measuring local heat flux, a thermal manikin has several important limitations. Firstly, it is difficult to arrange tight-fitting clothing on a stiff manikin with a limited number of joints. Any free spaces filled with air, resulting from, e.g., a twisted sleeve, influence heat flux and may result in differences in local thermal insulation between symmetrically located manikin segments. To prevent such effects in the future, at least three independent tests should be performed, with the manikin fully dressed and undressed each time. This is particularly important in tests of heated clothing, where heat is delivered by both the thermal manikin and the garment.

Moreover, a Newton-type manikin does not simulate blood circulation which contributes to heat distribution. Its fitness for investigating the effects of varied physical activities of wearers is also extremely limited. Finally, it does not represent the diversity of human bodies. Therefore, thermal performance of the developed clothing ensemble should be additionally assessed in tests with human participants, involving mountain rescuers as the intended end users and including their subjective perceptions which may differ to some extent from objective measures [19]. This will be another subject of further work.

## Figures and Tables

**Table 1:** Structures and materials of the tested garments.

Clothing Layer	Garment	Structure	Composition	Surface Mass, g/m^2^	Thermal Resistance, m^2^K/W	Water Vapor Resistance, m^2^Pa/W
1	Heated suit	Knitted microfleece fabric incorporating conductive thread	Fabric: 100% PES Heating thread: 100% stainless steel (diameter: 0.3 mm, resistance: ca. 30 Ω/m)	Knitted fabric: 242 ± 2	0.066 ± 0.000	5.93 ± 0.38
2	Down jacket	Down package: fabric + filling + fabric; X-type chambers 8.5 cm wide	Fabric: 100% PA Filling: 92% goose down, 8% feathers (elasticity: 750 in^3^)	Woven fabric (without filling): 37 ± 1	0.464 ± 0.018	50.0 ± 1.5
3	Outer suit	Three-layer laminate	100% PA + 100% PU (waterproof membrane) + 100% PA	Laminate: 142 ± 2	0.017 ± 0.002	14.50 ± 0.35

Acronyms used: PA (polyamide), PES (polyester), PU (polyurethane).

**Table 2:** Tested clothing ensemble configurations.

Symbol	Meaning	Heated Suit	Down Jacket	Outer Suit	Heating Power
REF	Reference	Yes	No	Yes	Off
DJ	Down jacket (unheated)	Yes	Yes	Yes	Off
HL	Heated, low power	Yes	No	Yes	Low
HH	Heated, high power	Yes	No	Yes	High

**Table 3:** Relative (percentages) and absolute (watts) heating powers applied during the tests.

Configuration	Back	Arms	Abdomen	Legs	Total
REF	0% (0.0 W)	0% (0.0 W)	0% (0.0 W)	0% (0.0 W)	0% (0.0 W)
DJ	0% (0.0 W)	0% (0.0 W)	0% (0.0 W)	0% (0.0 W)	0% (0.0 W)
HL	20% (3.4 W)	20% (3.4 W)	20% (1.7 W)	20% (1.7 W)	20% (10.3 W)
HH	25% (4.8 W)	75% (12.9 W)	30% (2.6 W)	100% (8.6 W)	55% (28.4 W)

**Table 4:** Clothing pre-treatment procedures according to [32], and process parameters.

Garment	Washing			Drying	
	Procedure	Temperature	Agent	Procedure	Process
Heated suit	3N	30 °C	Mild detergent	A	Open-air line dry
Down jacket	3N	30 °C	Mild detergent	F	Tumble dry at 60 °C
Outer suit	4N	40 °C	Reference detergent according to EN ISO 6330 [31]	A	Open-air line dry

**Table 5:** Thermal manikin segments and regions (see Figure 5) considered in this work.

No.	Designation	Description	Body Region
3	R Up Arm Fr	Right arm, front	Upper Limbs
4	R Up Arm Bk	Right arm, back	
5	L Up Arm Fr	Left arm, front	
6	L Up Arm Bk	Left arm, back	
7	R Forearm Fr	Right forearm, front	
8	R Forearm Bk	Right forearm, back	
9	L Forearm Fr	Left forearm, front	
10	L Forearm Bk	Left forearm, back	
11	R Hand	Right hand	
12	L Hand	Left hand	
13	Upper Chest	Chest, upper part	Torso
14	Shoulders	Back, upper part	
15	Stomach	Torso, middle part (abdomen)	
16	Mid-Back	Back, middle part	
17	Waist	Waist	
18	Lower Back	Back, lower part	
19	R Up Thigh Fr	Right thigh, upper part, front	Lower Limbs
21	R Up Thigh Bk	Right thigh, upper part, back	
22	L Up Thigh Fr	Left thigh, upper part, front	
24	L Up Thigh Bk	Left thigh, upper part, back	
25	R Lwr Thigh Fr	Right thigh, lower part, front	
26	R Lwr Thigh Bk	Right thigh, lower part, back	
27	L Lwr Thigh Fr	Left thigh, lower part, front	
28	L Lwr Thigh Bk	Left thigh, lower part, back	
29	R Calf Fr	Right calf, front	
30	R Calf Bk	Right calf, back	
31	L Calf Fr	Left calf, front	
32	L Calf Bk	Left calf, back	
33	L Foot	Right foot	
34	R Foot	Left foot	

**Table 6:** Measured thermal insulation of the tested clothing ensemble.

Clothing Configuration	Total Thermal Insulation, I_t_		Basic Thermal Insulation, I_cl_		Resultant Effective Thermal Insulation, I_cler_	
	Absolute, m^2^K/W	Relative Difference	Absolute, m^2^K/W	Relative Difference	Absolute, m^2^K/W	Relative Difference
REF	0.275	-	0.222	-	0.183	-
DJ	0.367	33%	0.320	44%	0.267	46%
HL	0.305	11%	0.254	15%	0.211	15%
HH	0.379	38%	0.333	50%	0.279	52%

**Table 7:** Key parameters of this and similar solutions of heated clothing.

Reference	Main Clothing Layers (Inner to Outer)	Heating Pads					Thermal Insulation with Heating	
		Materials	Quantity	Locations	Total Area, cm^2^	Total Power Applied, W	Absolute, m^2^K/W	Increase Relative to No Heating
Wang and Lee [27]	1. Shirt 2. Vest * 3. Jacket	Carbon polymer fabric	1	Back (1)	500	13	ca. 0.26 (0 °C and −10 °C)	2%
Song et al. [29]	1. Underwear 2. Outerwear 3. Vest *	Carbon wire between PES fabrics	7	Back (3) Waist Kneecaps	2212	26.5	0.365 (2 °C)	11%
Li et al. [26]	1. Sweater 2. Jacket *	Graphene sheet	8	Back Chest Abdomen Upper arms	1400	25.2	0.250 ** (−5 °C to −15 °C; 0.5 m/s)	7% **
This work	1. Inner suit * 2. Outer suit	Steel thread sewn onto PES fabric	12	Shoulders Lower back Waist Upper arms Lower arms Lower thighs	840	10.3	0.305 0.254 ** (−7 °C)	11% 15% **
						28.4	0.379 0.333 ** (−7 °C)	38% 50% **

Heating pads: two in each location unless indicated otherwise. Thermal insulation: total insulation, I_t_, at the air velocity of 0.4 m/s unless indicated otherwise; air temperature during tests shown in parentheses. * Heated layer. ** Basic insulation, I_cl_ (ambiguous in [26], see the discussion in text).

## Data Availability

The original contributions presented in this study are included in the article. Further inquiries can be directed to the corresponding author.

## Abbreviations

The following abbreviations are used in this manuscript:

AgNW	Silver nanowire
CHG	Chemically heated garment
CNT	Carbon nanotube
EHG	Electrically heated garment
GPS	Global Positioning System
NA	Not applicable
PA	Polyamide
PES	Polyester
PU	Polyurethane

## References

[1] Lee H., Baek K. (2021). Developing a smart multifunctional outdoor jacket with wearable sensing technology for user health and safety. Multimed. Tools Appl..

[2] Kim K., Kim S., Lim D., Ha J., Jeong W. (2021). Analysis of design elements and heating system of domestic and foreign commercial electrical heated clothing. Fash. Text. Res. J..

[3] Lee B., Lee J. (2015). Development of design for band type heating vests. J. Fash. Bus..

[4] Fang S., Wang R., Ni H., Liu H., Liu L. (2020). A Review of flexible electric heating element and electric heating garments. J. Ind. Text..

[5] Lee J., Lee B. (2014). Development of design for heating vest with detachable heating device. J. Fash. Bus..

[6] Lee J. (2018). A Study of the analysis on the risk of ignition and low-temperature burns caused by the use of electrically heated clothes. J. Soc. Disaster Inf..

[7] Repon M.R., Mikučionienė D. (2021). Progress in flexible electronic textile for heating application: A critical review. Materials.

[8] Xu P., Wang F., Zhao M., Wang F., Gao C. (2014). Electrically heated clothing (ehc) for protection against cold stress. Protective Clothing.

[9] Choi H.N., Jee S.H., Ko J., Kim D.J., Kim S.H. (2021). Properties of surface heating textile for functional warm clothing based on a composite heating element with a positive temperature coefficient. Nanomaterials.

[10] Zhang Y., Wang H., Lu H., Li S., Zhang Y. (2021). Electronic fibers and textiles: Recent progress and perspective. iScience.

[11] Wang F., Gao C., Kuklane K., Holmér I. (2010). A Review of technology of personal heating garments. Int. J. Occup. Saf. Ergon..

[12] Kayacan O., Yazgan Bulgun E. (2009). Heating behaviors of metallic textile structures. Int. J. Cloth. Sci. Technol..

[13] Yang H. (2017). Research on application of carbon fiber heating material in clothing. IOP Conf. Ser. Earth Environ. Sci..

[14] Hsu P.-C., Liu X., Liu C., Xie X., Lee H.R., Welch A.J., Zhao T., Cui Y. (2015). Personal thermal management by metallic nanowire-coated textile. Nano Lett..

[15] Doganay D., Coskun S., Genlik S.P., Unalan H.E. (2016). Silver nanowire decorated heatable textiles. Nanotechnology.

[16] Cho H., Cho S.W. (2015). Optimal heating location for developing the heating smart clothing based on thermal response of body. Sci. Emot. Sensib..

[17] Castellani J.W., Yurkevicius B.R., Jones M.L., Driscoll T.J., Cowell C.M., Smith L., Xu X., O’Brien C. (2018). Effect of localized microclimate heating on peripheral skin temperatures and manual dexterity during cold exposure. J. Appl. Physiol..

[18] Kim S., Park D., Lim D., Yoo E.-S., Lee J.-Y., Kong Y.-K., Jeong W. Influence of the forearm or hand heating on skin temperature, thermal sensation, and manual dexterity during cold exposure (−5 °C): A pilot study. Proceedings of the Autumn Conference of the Ergonomics Society of Korea.

[19] Kurczewska A., Leśnikowski J. (2008). Variable-thermoinsulation garments with a microprocessor temperature controller. Int. J. Occup. Saf. Ergon..

[20] Kayacan O., Bulgun E., Sahin O. (2009). Implementation of steel-based fabric panels in a heated garment design. Text. Res. J..

[21] Liu H., Zhao H., Li X., Wu L., Chen X., Zhang M. (2014). Flexible Heating Fabric System. CN Patent.

[22] Lee E., Roh J.-S., Kim S. (2018). User-centered interface design approach for a smart heated garment. Fibers Polym..

[23] Fan X., Lin H., Ye C., Guo Y., Huang L. Smart heating clothes based on bluetooth. Proceedings of the 2019 14th International Conference on Computer Science & Education (ICCSE).

[24] Lee H., Hong K., Lee Y., Kim S. (2017). User’s voluntary heating behavior for the programming of the efficient heating mode of smart base layer clothing. J. Korean Soc. Cloth. Text..

[25] (2004). Clothing—Physiological Effects—Measurement of Thermal Insulation by Means of a Thermal Manikin.

[26] Li S., Deng Y., Cao B. (2023). Study on the performance of personal heating in extremely cold environments using a thermal manikin. Buildings.

[27] Wang F., Lee H. (2010). Evaluation of an electrically heated vest (EHV) using a thermal manikin in cold environments. Ann. Occup. Hyg..

[28] (2017). Protective Clothing—Ensembles and Garments for Protection Against Cold.

[29] Song W., Lai D., Wang F. (2015). Evaluating the cold protective performance (CPP) of an electrically heated garment (EHG) and a chemically heated garment (CHG) in cold environments. Fibers Polym..

[30] Park J.H. (2016). Smart Heating Clothes, System and Method for Controlling Heating Thereof.

[31] Pękosławski B., Marciniak P., Starzak Ł., Stawiński A., Bartkowiak G. (2022). Power supply and control unit for actively heated protective clothing with photovoltaic energy harvesting. Energies.

[32] (2021). Textiles—Domestic Washing and Drying Procedures for Textile Testing.

[33] Młynarczyk M., Orysiak J., Jankowski J. (2025). Effect of workwear fit on thermal insulation: Assessment using 3d scanning technology. Materials.

[34] (2004). Protective Clothing—Garments and Clothing Combinations for Protection Against Cold.

